# The preventive and therapeutic effects of AAV1‐KLF4‐shRNA in cigarette smoke‐induced pulmonary hypertension

**DOI:** 10.1111/jcmm.16194

**Published:** 2020-12-20

**Authors:** Desheng Sun, DanDan Ding, Qinghai Li, Min Xie, Yongjian Xu, Xiansheng Liu

**Affiliations:** ^1^ Department of Respiratory and Critical Care Medicine Key Laboratory of Pulmonary Diseases of Health Ministry Tongji Hospital Tongji Medical College Huazhong University of Science and Technology Wuhan China; ^2^ Department of Respiratory and Critical Care Medicine Affiliated Hospital of Zunyi Medical University Zunyi China

**Keywords:** AAV1, cigarette smoke, KLF4, prevention, pulmonary hypertension, therapeutic efficacy

## Abstract

We found previously that KLF4 expression was up‐regulated in cultured rat and human pulmonary artery smooth muscle cells (PASMCs) exposed to cigarette smoke (CS) extract and in pulmonary artery from rats with pulmonary hypertension induced by CS. Here, we aim to investigate whether CS‐induced pulmonary hypertension (PH) is prevented and ameliorated by targeted pulmonary vascular gene knockdown of KLF4 via adeno‐associated virus 1 (AAV1)‐KLF4‐shRNA in vivo *in rat model*. The preventive and therapeutic effects were observed according to the different time‐point of AAV1‐KLF4‐shRNA intratracheal administration. We tested haemodynamic measurements of systemic and pulmonary circulations and observed the degree of pulmonary vascular remodelling. In the preventive experiment, KLF4 expression and some pulmonary circulation hemodynamic measurements such as right ventricular systolic pressure (RVSP), mean right ventricular pressure (mRVP), peak RV pressure rate of rise (dP/dt max) and right ventricle (RV) contractility index were increased significantly in the CS‐induced PH model. While in the prevention group (AAV1‐KLF4‐shRNA group), RVSP, mRVP, dP/dt max and RV contractility index which are associated with systolic function of right ventricle decreased and the degree of pulmonary vascular remodelling relieved. In the therapeutic experiment, we observed a similar trend. Our findings emphasize the feasibility of sustained pulmonary vascular KLF4 gene knockdown using intratracheal delivery of AAV1 in an animal model of cigarette smoke‐induced PH and determined gene transfer of KLF4‐shRNA could prevent and ameliorate the progression of PH.

## 1 INTRODUCTION

Pulmonary hypertension (PH) is a kind of devastating vascular diseases with significant characteristics of vasoconstriction and vascular remodelling, which finally results in right ventricular failure (RVF) and death.^1^, ^2^, ^3^ Although some pharmacological agents have been developed for treating PH in recent years, the long‐term prognosis of patients with serve PH remains poor. Most therapeutic approaches target to combating the aetiology. In pulmonary arterial hypertension (PAH, WHO group 1 PH), the current treatments mainly aim to induce pulmonary artery vasodilation and ease right ventricular after‐load, rather than target to vascular remodelling and ameliorate the excess arterial smooth muscle cells accumulation. So, the long‐term treatment effects were limited. So far, the underlying mechanism of PH and vascular remodelling is not well defined, especially about the key cellular and molecular events and signalling pathways underlying the vessel hypermuscularization.^4^, ^5^

Cigarette smoke (CS) is a known risk factor for pulmonary hypertension,^6^ which can directly cause remodelling of pulmonary small vessels.^7^, ^8^, ^9^, ^10^, ^11^ Some studies also found that smokers in some chronic obstructive pulmonary disease (COPD) patients had small pulmonary vascular remodelling in the early stage of disease.^7^, ^12^, ^13^ Our previous work suggested that Krüppel‐like factor 4 (KLF4) play an important role in the promotion of PASMC proliferation and migration and vascular remodelling induced by CS, which was the key pathogenesis of PH.^14^ With recombinant adeno‐associated virus (AAV) vectors, current research aims to observe whether the KLF4 gene knockdown in the pulmonary vessels has a significant inhibition or remission effect on the development of PH in vivo. We therefore suggested that gene knockdown of KLF4 via intratracheal administration of AAV1‐KLF4‐shRNA would provide selective gene silencing to the pulmonary circulation to prevent or ameliorate pulmonary vascular remodelling and right ventricular hemodynamic consequences in CS‐PH.

We also evaluated the feasibility of sustained pulmonary vascular KLF4 gene silencing in a rat model of CS‐PH and to assess the efficiency of gene knockdown on the development of pulmonary vessel and right ventricular remodelling. On the basis of previous research results,^15^ we used a new intratracheal injection gene silencing method for least off‐target transgene expression and most safety.

## 2 MATERIALS AND METHODS

### 2.1 Ethics statement

Homozygous adult male SD rats weighing 185 to 225 g (8 weeks old) were obtained from Hubei Research Center of Laboratory Animal, China. This study was approved by the Animal Experimentation Ethics Committee of Tongji Medical College, Huazhong University of Science and Technology, Wuhan, China.

### 2.2 Preparation of AAV1‐KLF4‐shRNA

A U6 promoter‐driven shRNA expression system was established in an AAV1 vector. Green fluorescent protein (GFP) expression was separately controlled by a CMV promoter as a marker for transduction efficiency. KLF4 shRNA was designed based on the siRNA sequence (GenBank Acc. NM_053713.1) and was screened according to the guidelines reported.^16^ Three selected siRNA target sequences were inserted between the BamHI and EcoRI sites in a U6‐CMV‐EGFP/ AAV vector, and an optimal KLF4 target (sequence: 5′‐ CACCCACACTTGTGACTAT −3′) was selected. A recombinant adenovirus carrying a siRNA sequence targeting the eGFP reporter gene (sequence: 5′‐ TTCTCCGAACGTGTCACGTAA −3′) was included as a control. Both the adenovirus‐KLF4‐shRNA and negative control vectors contained the sequence encoding GFP. All constructs were verified by DNA sequencing, all viral vectors were generated by triple plasmid cotransfection of human 293 cells, and recombinant virions were column purified. Next, viral titres were determined using qPCR.^17^ The resulting AAV1‐KLF4‐shRNA titre was determined to be 1.2 × 10^12^ vector genomes (vg)/ml, and the AAV1‐GFP titre was 1.5 × 10^12^ vg/ml. During the follow‐up injection, 125 μl was injected into each rat in AAV1‐KLF4‐shRNA group and 100 μl in AAV1 control vector group.

### 2.3 An animal model of pulmonary hypertension induced by cigarette smoke

A rat model of CS‐induced PH was established as described previously.^14^, ^18^ The smoking group underwent whole body exposure to CS in a ventilated chamber, as described previously.^19^ The smoking group received 1‐hour CS exposure of 12 cigarettes two times per day, every day for 4 months. Cigarettes (HongJinLong, Wuhan Tobacco Company, China; 9 mg tar, 0.6 mg nicotine and 11 mg carbon monoxide in each cigarette) were used. During the whole experimental time, the oxygen concentration in the chamber ranged from 18% to 21%, and the carbonic oxide concentration ranged from 800 to 1000 ppm.

### 2.4 Study design

**FIGURE 1:**
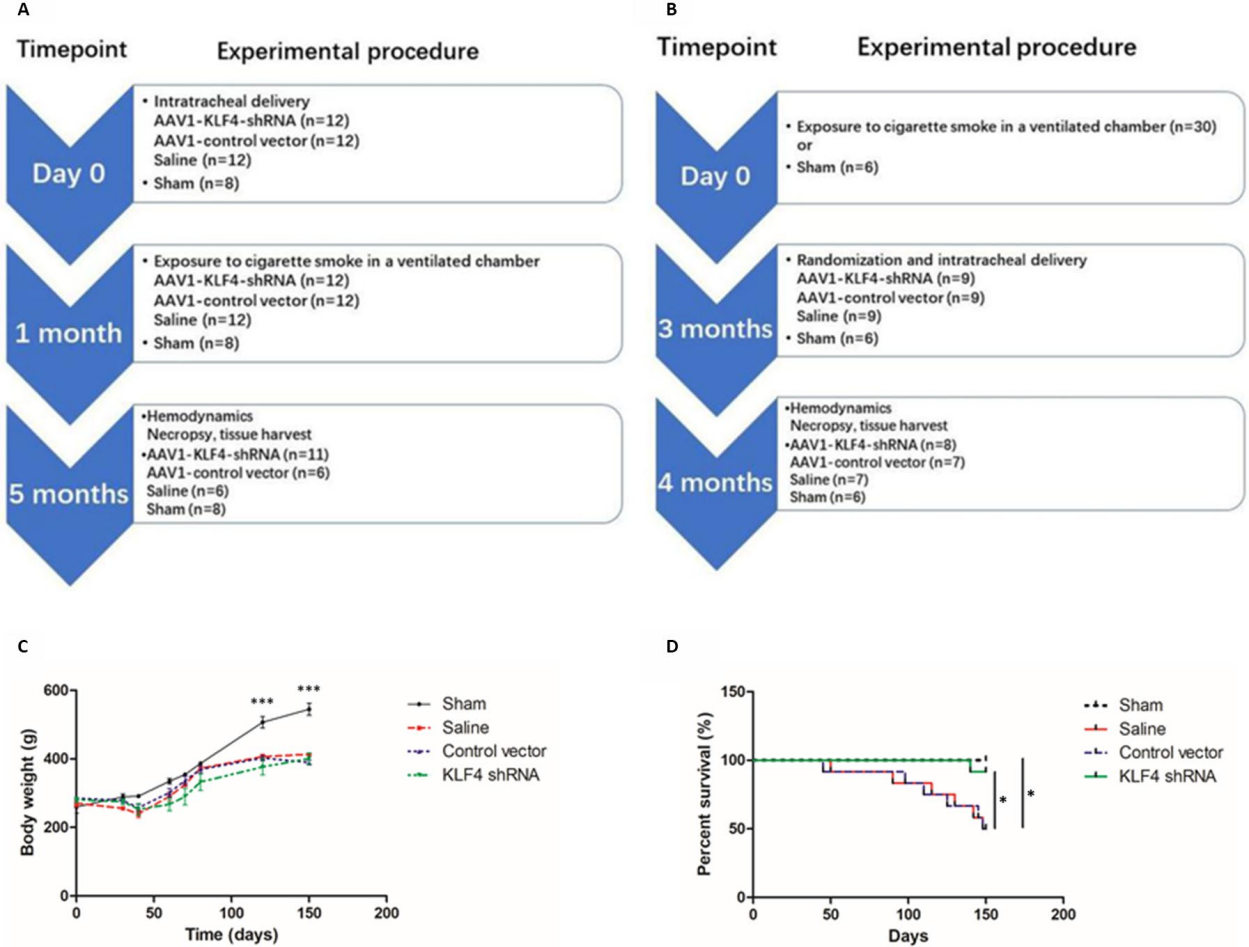
Study Design and Timeline of the prevention experiment (A) and the therapeutic experiment (B). Curve of body weight change (C) and survival curve (D) of the prevention experiment. Multiple comparisons were performed by one‐way ANOVA with SNK‐q, and log‐rank test was used to compare the survival curves. *P < .05, ***P < .001

In the prevention protocol, 44 rats were randomly assigned to one of four groups: sham (n = 8), saline (n = 12), AAV1‐control vector (n = 12) or AAV1‐KLF4‐shRNA (n = 12). The latter three groups were administrated by intratracheal injection of saline, AAV1 carrying control vector or AAV1 carrying the KLF4‐shRNA as we described previously.^20^ 30 days after the intratracheal administration, the latter three groups underwent whole body exposure to CS for another 4 months. The sham group was exposed to air as a control for the PH model. Haemodynamic studies were conducted 4 months after the CS or air exposure after which the rats were killed for tissue collection. The specific experimental process and time‐points refer to Figure 1A.

In the therapeutic protocol, 36 rats were randomly assigned to two groups: sham (n = 6) and smoke (n = 30). 3 months after the stimulation of cigarette smoke, 27 rats in the smoking group (3 died in the original group) were randomly divided into three groups: saline (n = 9), AAV1‐control vector (n = 9) or AAV1‐KLF4‐shRNA (n = 9) and were administrated by intratracheal injection of saline, AAV1 carrying control vector or AAV1 carrying the KLF4‐shRNA, respectively. These three groups were continued for 1 month according to the above cigarette smoke stimulation method. The rats in the sham group did not get any treatment. After completion of the model, haemodynamic indexes were measured in anaesthetized rats, so as to preliminarily evaluate the construction effect and intervention effect of the pulmonary hypertension model. The specific experimental process and time‐points refer to Figure 1B.

### 2.5 Detection of pulmonary hemodynamic indexes

Right ventricle systolic pressure (RVSP) was measured in anaesthetized rats through an abdominal incision, as previously described.^21^, ^22^, ^23^, ^24^ The rats were anaesthetized using 2% sodium pentobarbital (40 mg/kg), and the diaphragm was visualized through the abdomen. RV pressure was measured using a needle filled with heparinized saline and connected to a pressure transducer. The pressure was recorded using Power Lab Software (ADI Instruments). Only the rats from which stable tracings were obtained, and the RV punctures was verified were included in the analysis. The left ventricular pressure was measured in the same way. The heart rate, mean right ventricular pressure (mRVP), right ventricular peak RV pressure rate of rise (dP/dt max), peak RV pressure rate of decline (dP/dt min), time constant of isovolumic relaxation (Tau), RV contractility index, right and left ventricular end‐systolic and diastolic pressures were measured directly. Correct localization of the puncture was verified although post‐mortem inspection.

### 2.6 Calculation of Fulton's index

Hearts were filled with 0.9% saline and followed by constant‐pressure infusion of saline for half an hour with needle inserted into the left ventricle and a small hole in the right atrium. This can remove remnant blood. The atria/valves and extraneous vascular material were removed from the heart. The free wall of the RV was dissected from the left ventricle (LV) and septum (S), and both portions were quickly blotted dry. RV weight, RV weight/body weight (RV/BW) and Fulton's index (a weight ratio of [RV / (LV + S)]) were calculated to determine right ventricular hypertrophy.

### 2.7 Pulmonary vascular remodelling

Alterations in pulmonary vessels were measured by HE staining. Pulmonary vessels with an external diameter ranging from 20 to 150 μm were chosen for assessment. Seven arteries per rat were selected, and medial wall thickness was calculated.

### 2.8 Immunochemistry staining

The extent of muscularization of small arteries was assessed following immunochemistry staining for α‐smooth muscle actin (α‐SMA) (Abcam, Cambridge, UK). Meanwhile, smooth muscle cells of small lung arteries exhibiting proliferation were assessed by immunostaining with proliferating cell nuclear antigen (PCNA) (ProteinTech, Wuhan, China) and osteopontin (OPN) (ProteinTech, Wuhan, China). Furthermore, the expression of KLF4 and P‐AKT was assessed by immunostaining with antibodies against KLF4 (ProteinTech, Wuhan, China) and P‐AKT (Gene Tex, San Antonio, USA).

### 2.9 qRT‐PCR

Total RNA was extracted from pulmonary arteriole with TRIzol reagent (Invitrogen, Thermo Fisher Scientific, US). cDNA was synthesized with a Prime Script RT Reagent Kit (TaKaRa Bio, Dalian, China). KLF4 mRNA expression was determined using gene‐specific primers and SYBR Green 1 with a Bio‐Rad iQ5 Multicolor Real‐Time PCR machine (Bio‐Rad, Hercules, US). The primers used in this study were as follows: KLF4: forward: 5′‐ TCACATGAAGCGACTTCCCC −3′, reverse, 5′‐ CGTTGAACTCCTCGGTCTCC −3′; β‐actin: forward, 5′‐ TCACCCACACTGTGCCCCATCTACGA‐3′, reverse, 5′‐CAGCGGAACCGCTCATTGCCAATGG −3′

### 2.10 Western blot

Total proteins were extracted from rat pulmonary arteries or PASMCs, and their concentrations were measured with a BCA kit (Servicebio, Wuhan, China). Primary antibodies against GAPDH (ProteinTech, Wuhan, China), KLF4 (ProteinTech, Wuhan, China) and PCNA (ProteinTech, Wuhan, China) were used. Bands were detected by a ChemiDoc MP System (Bio‐Rad Laboratories, Hercules, USA), and densitometry of Western blots was quantified using Image Lab software (Bio‐Rad, Hercules, USA).

### 2.11 Statistical analyses

All quantitative data were represented as the mean ± SEM. Multiple comparisons were performed by one‐way ANOVA followed by Students‐Newman‐Keuls (SNK‐q) post hoc test, and log‐rank test was used to compare the survival curves. SPSS software (version 17.0) and GraphPad Prism 5 was used to analyse data and draw statistical charts. Difference is considered statistically significant at *P* < .05.

## 3 RESULTS

### 3.1 Prevention of cigarette smoke‐induced PH by intratracheal delivery of AAV1‐KLF4‐shRNA

#### 3.1.1 The general condition of rats in the prevention experiment

Compared with the control group, the smoking rats were inactive, their hair was yellow and their weight gain was slower. (Figure 1C). During the 5‐month modelling period, there were 6 died rats in the saline group and the AAV1‐control vector group, respectively, and 1 died rats in the AAV1‐KLF4‐shRNA group. All deaths occurred in 4 months receiving smoke stimulation. (Figure 1D).

#### 3.1.2 Evaluation of pulmonary vascular transduction effect by injection of adenovirus associated virus 1 via airway and the effect of KLF4 knockdown

**FIGURE 2:**
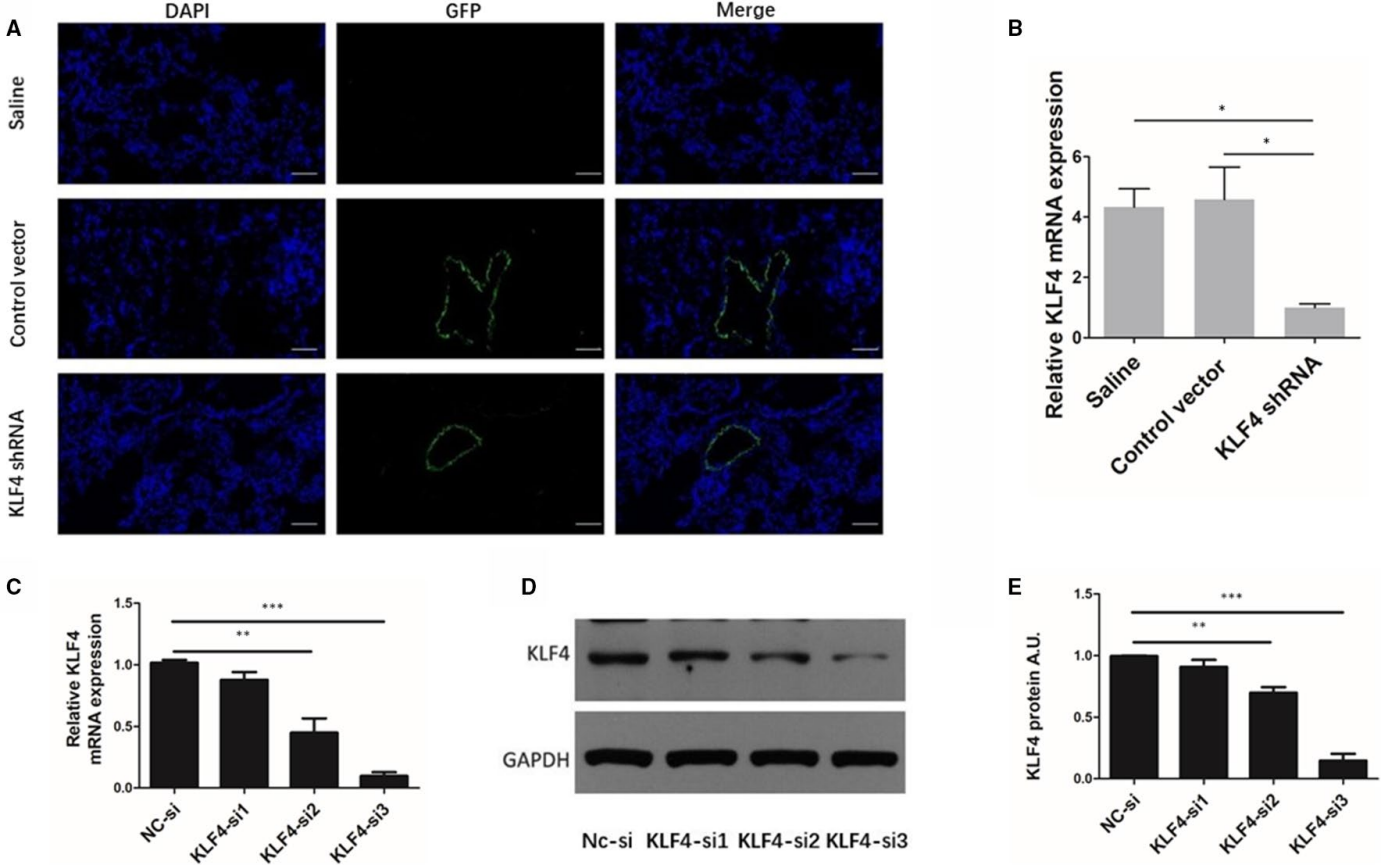
(A) The AAV1 (green fluorescence, GFP) expression in the pulmonary artery of rats; blue was DAPI dyed nucleus, and the scale bar: 30 µm. (B) the mRNA expression of KLF4 in rat pulmonary arterioles. Saline group (n = 6), AAV1‐control vector group (n = 6) and AAV1‐KLF4‐shRNA group (n = 11) at the end of the prevention experiment. The silencing effect of small interfering RNA on KLF4 in rat PASMCs was detected by real‐time fluorescent quantitative PCR (C) and Western blot (D, E). Relative protein levels of KLF4 were normalized to GAPDH. AU, arbitrary units. Multiple comparisons were performed by one‐way ANOVA with SNK‐q. *P < .05,**P < .01,***P < .001

As described in the method section, a sequence encoding GFP was inserted into the AAV structure. As shown in Figure 2A, the expression of green fluorescent protein (GFP) in the lungs of experimental animals was detected by fluorescence microscopy. To evaluate the distribution of target virus (AAV1‐KLF4‐shRNA) or control virus (AAV1‐control vector), as shown in the green fluorescence of the map, the AAV1 vectors were injected into the rat airway. Both the target virus and the control virus are distributed along the pulmonary vessels, which indicated that the transduction effect was good. To further determine the efficiency of KLF4 gene knockdown, we detected the mRNA expression of KLF4 in the pulmonary arterioles of three groups of rats under the same smoke stimulation by qRT‐PCR. The results showed that the target virus (AAV1‐KLF4‐shRNA) group was significantly lower than the other two groups (Figure 2B), indicating that the pulmonary vascular‐specific KLF4 knockdown model was successfully constructed. We also verified the effect of KLF4 knockdown at the cellular level. The silencing effect of small interfering RNA on KLF4 in rat PASMCs was detected by real‐time fluorescent quantitative PCR (Figure 2C) and Western blot (Figure 2D, 2E).

#### 3.1.3 CS‐induced rat pulmonary hypertension model was successfully constructed and preventive intervention of AAV1‐KLF4‐shRNA is effective

**FIGURE 3:**
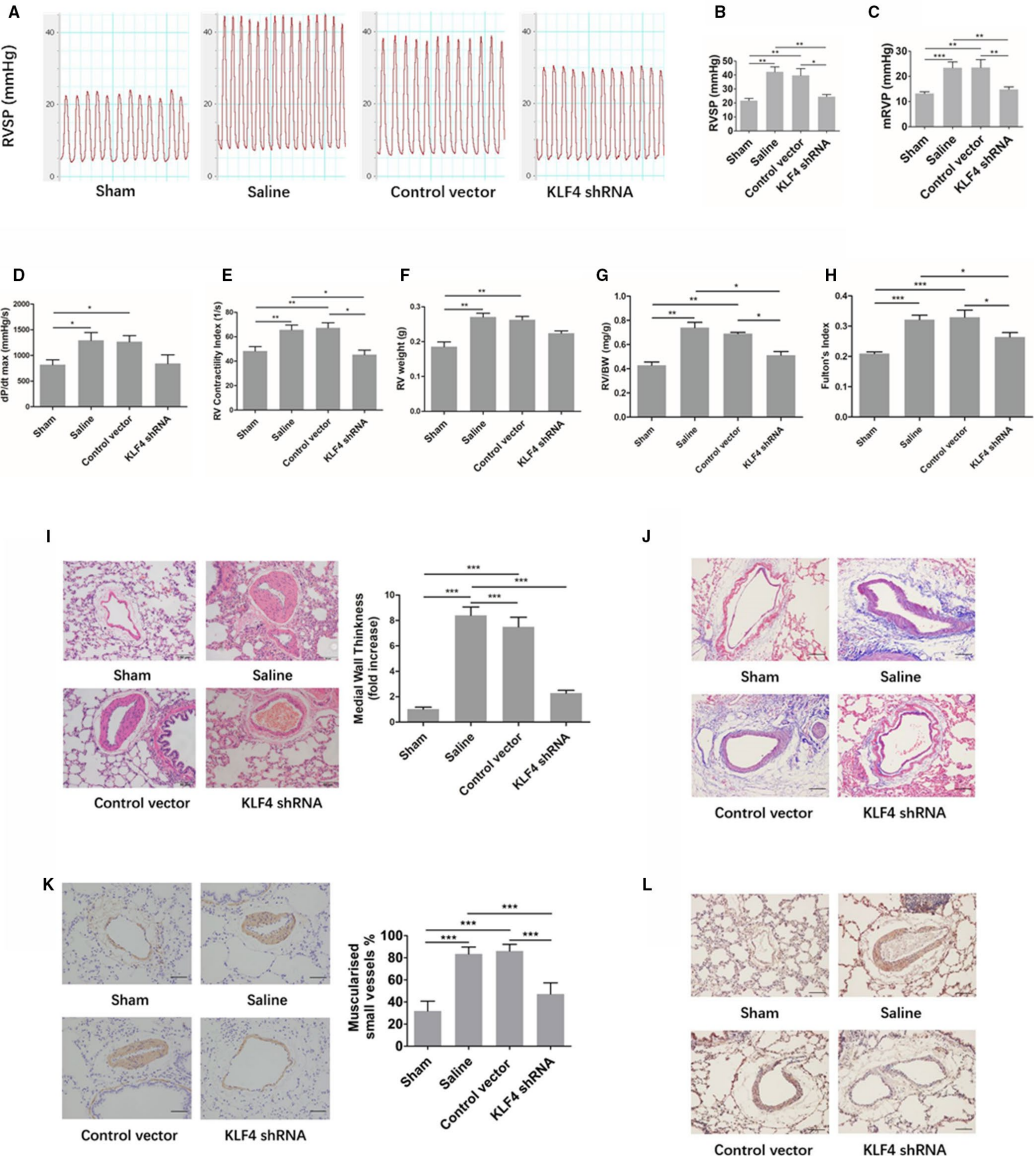
(A) The waveform diagram of the systolic pressure of the right ventricle. (B) A statistical map of right ventricular systolic pressure (C) A statistical map of the mean pressure of the right ventricle. The maximal increase rate of right ventricular pressure (dP/dt max) (D) and right ventricular systolic index (RV contractility index) (E) of each group. Right ventricular weight (RV weight) (F), right ventricular weight / body weight (RV/BW) (G) and right ventricular hypertrophy index (Fulton's index) (H). (I) HE staining of pulmonary vessels and comparison of medial wall thickness in pulmonary arterioles. Scale bar: 50 μm (J)Masson staining of collagen around rat pulmonary arterioles. Scale bar: 20 μm (K) Comparison of the degree of pulmonary vascularized in rats of each group. Scale bar: 20 μm (L) Expression of OPN in the pulmonary vessels of rats in each group. Scale bar: 30 μm. Sham (n = 8), saline (n = 6), AAV1‐control vector group (n = 6) and AAV1‐KLF4‐shRNA group (n = 11) at the end of the prevention experiment. Multiple comparisons were performed by one‐way ANOVA with SNK‐q. *P < .05,**P < .01,***P < .001

As shown in Figure 3A‐3C, the right ventricular systolic pressure (RVSP) and the right ventricular mean pressure (mRVP) in the saline group and the AAV1‐control vector group were significantly higher than those in the sham group, which suggest that the model of pulmonary hypertension was constructed successfully. In the AAV1‐KLF4‐shRNA group, RVSP and mRVP were significantly lower than those in the saline group and the AAV1‐control vector group, indicating that preventive interventions were effective.

#### 3.1.4 Other indicators reflect the contractile and diastolic functions of the right ventricle in the prevention experiment

As shown in Figure 3D and Figure 3E, the peak RV pressure rate of rise (dP/dt max) and right ventricular systolic index (RV contractility index) in the saline group and the AAV1‐control vector group were significantly higher than those in the sham group. In the AAV1‐KLF4‐shRNA group, the RV contractility index was significantly lower than that in the saline group and the AAV1‐control vector group, while dP/dt Max had a downward trend compared with the two groups of PH model, but the difference was not statistically significant. The right ventricular end diastolic pressure (RVEDP), the peak RV pressure rate of decline (dP/dt min) and the right ventricle time constant of isovolumic relaxation (Tau) were not significantly different among each group (Figure_1_SuppInfo).

#### 3.1.5 Left ventricular hemodynamic parameters and heart rate changes in the prevention experiment

As shown in Figure_2_SuppInfo, there was no significant difference between the left ventricular end systolic pressure (LVESP) and left ventricular end diastolic pressure (LVEDP). The heart rate in all groups was within 271‐332/ minute range, and the difference of heart rate between each group was not statistically significant, so the haemodynamic test data of each group were comparable.

#### 3.1.6 Changes in the level of right ventricular hypertrophy in the prevention experiment

As shown in Figure 3F‐3H, the indices of right ventricular hypertrophy, such as right ventricular weight (RV weight), right ventricular hypertrophy index (Fulton 's index) and right ventricular weight/ body weight (RV/BW), were significantly higher in the saline group and the AAV1‐control vector group than in the sham group. The RV weight in the AAV1‐KLF4‐shRNA group seemed lower than that in the saline group and the AAV1‐control vector group, but the difference was not statistically significant. The Fulton 's index and RV/BW in the AAV1‐KLF4‐shRNA group were significantly lower than those in the saline group and the AAV1‐control vector group.

#### 3.1.7 Alterations of pulmonary arteriole remodelling and muscularization in the prevention experiment

As shown in Figure 3I, compared with the sham group, the pulmonary small vascular wall of the saline group and the AAV1‐control vector group were thickened, and their smooth muscle of the middle layer was significantly proliferated. In the AAV1‐KLF4‐shRNA group, the thickening of the small vascular wall was significantly reduced compared with that in the saline group and the AAV1‐control vector group. Similarly, Masson staining showed that the deposition of matrix protein around pulmonary vessels in model group was increased, which was significantly reduced after preventive intervention (Figure 3J). As shown in Figure 3K, the muscularized degree of pulmonary vessels in the saline group and the AAV1‐control vector group was significantly higher than that in the sham group. The muscularized degree of pulmonary vessels in the AAV1‐KLF4‐shRNA group was significantly lower than that in the saline group and the AAV1‐control vector group.

#### 3.1.8 Changes of the expression of OPN, KLF4 and the number of PCNA‐positive cells in the pulmonary vessels in the prevention experiment

The expression of OPN in the pulmonary vessels of the saline group and the AAV1‐control vector group was significantly stronger than that in the sham group, but the expression of OPN in the pulmonary vessels of the AAV1‐KLF4‐shRNA group was significantly weaker than that of the saline group and the AAV1‐control vector group (Figure 3L).

**FIGURE 4:**
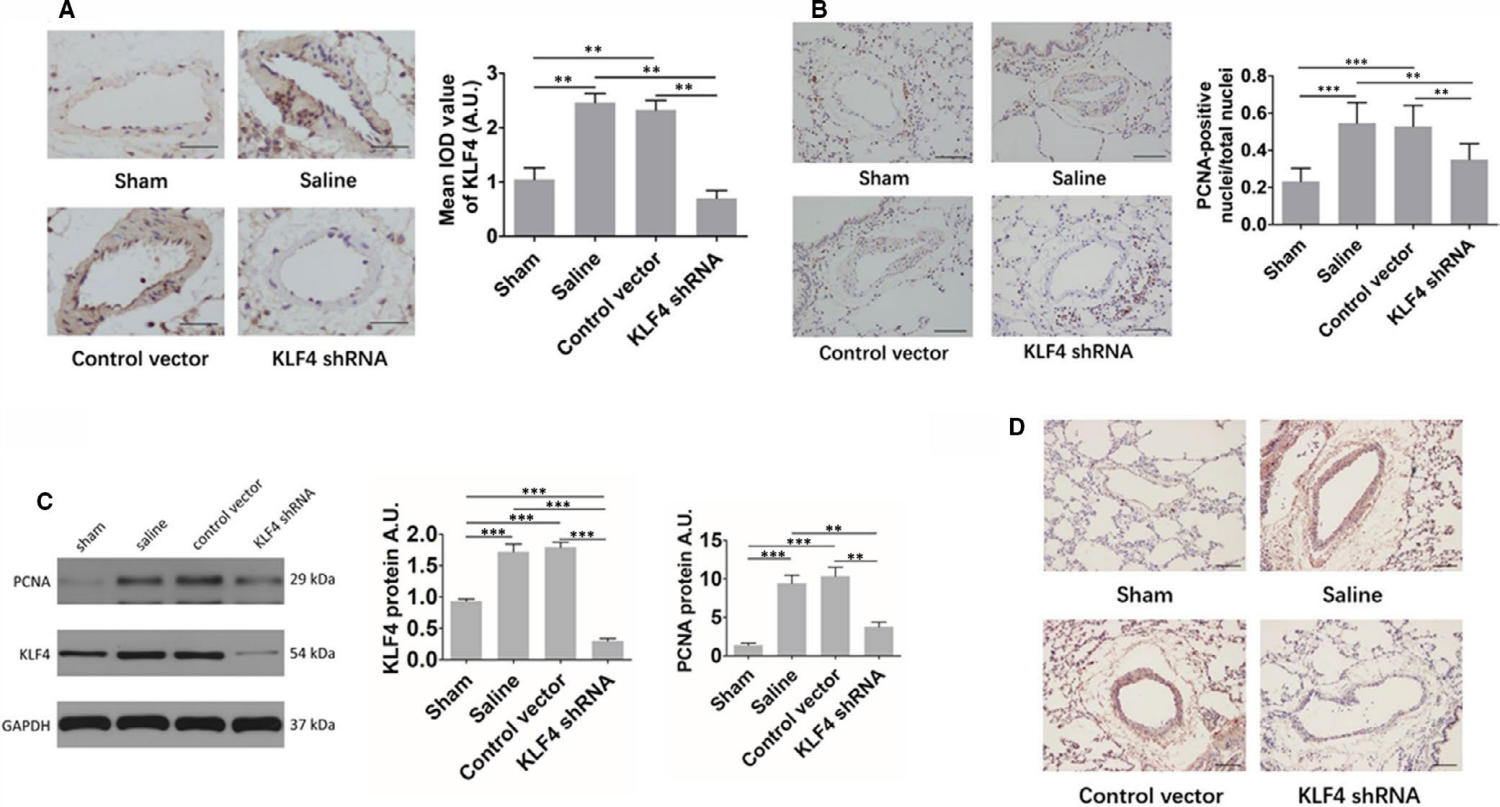
(A) Expression of KLF4 in the pulmonary vessels of rats in each group. Scale bar: 30 μm (B) Expression of PCNA in the pulmonary vascular smooth muscle of rats in each group. Scale bar: 25 μm (C) Representative bands of protein expression for KLF4 and PCNA in rat pulmonary vessels, and comparison of relative protein levels (normalized to GAPDH level). AU, arbitrary units. (D) Expression of P‐AKT in the pulmonary vessels of rats in each group. Scale bar: 20 μm. Sham (n = 8), saline (n = 6), AAV1‐control vector group (n = 6) and AAV1‐KLF4‐shRNA group (n = 11) at the end of the prevention experiment. Multiple comparisons were performed by one‐way ANOVA with SNK‐q. **P < .01, ***P < .001

The expression of KLF4 in the pulmonary vessels of the saline group and the AAV1‐control vector group was significantly stronger than that in the sham group, but the expression of KLF4 in the pulmonary vessels of the AAV1‐KLF4‐shRNA group was significantly weaker than that of the saline group and the AAV1‐control vector group (Figure 4A).

The immunohistochemical test of proliferating cell nuclear antigen(PCNA) showed that the amount of PCNA‐positive smooth muscle cells in small lung vessels of the saline group and the AAV1‐control vector group appeared to be much more than that of the sham group, but the amount of PCNA‐positive smooth muscle cells in small lung vessels of the AAV1‐KLF4‐shRNA group was significantly less than that of the saline group and the AAV1‐control vector group (Figure 4B).

We also detected the protein expression for KLF4 and PCNA in rat pulmonary vessels by Western blot. The results were consistent with the trend of immunohistochemistry (Figure 4C).

#### 3.1.9 Changes of the expression of P‐AKT in the pulmonary vessels in the prevention experiment

In our previous study, we found that KLF4 knockdown inhibited the CSE‐induced increase of AKT phosphorylation in PASMCs.^14^ Therefore, in this study, we detected the expression of P‐AKT in pulmonary vessels of rats in each group. As shown in Figure 4D, The expression of P‐AKT in the pulmonary vessels of the saline group and the AAV1‐control vector group was significantly stronger than that in the sham group, but the expression in the pulmonary vessels of the AAV1‐KLF4‐shRNA group was significantly weaker than that of the saline group and the AAV1‐control vector group.

### 3.2 Therapeutic experiment of AAV1‐ KLF4‐shRNA in Cigarette Smoke‐Induced Pulmonary Hypertension

Given that AAV1‐KLF4‐shRNA could prevent the development of CS‐induced PH, we also sought to determine whether gene knockdown of KLF4 via AAV1‐KLF4‐shRNA had therapeutic efficacy in established PAH. To examine this hypothesis, we administered intratracheally saline, AAV1‐control vector or AAV1‐KLF4‐shRNA after 3 months of cigarette smoke stimulation. (Figure 1B).

#### 3.2.1 Indicators reflect the contractile function of the right ventricle in the therapeutic experiment

**FIGURE 5:**
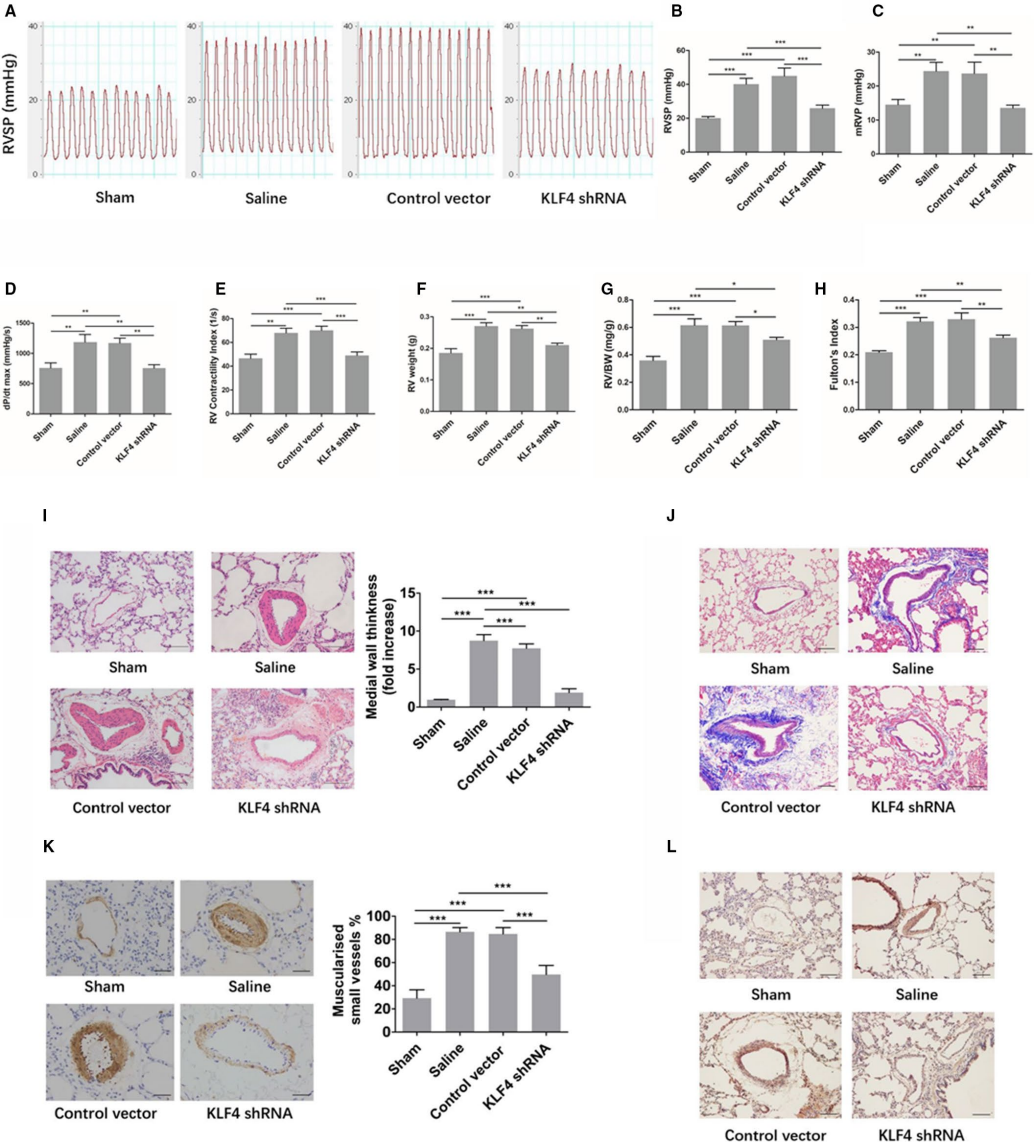
(A) The waveform diagram of the systolic pressure of the right ventricle. (B) A statistical map of right ventricular systolic pressure (C) A statistical map of the mean pressure of the right ventricle. The maximal increase rate of right ventricular pressure (dP/dt max) (D) and right ventricular systolic index (RV contractility index) (E) of each group. Right ventricular weight (RV weight) (F), right ventricular weight / body weight (RV/BW) (G) and right ventricular hypertrophy index (Fulton's index) (H). (I) HE staining of pulmonary vessels and comparison of medial wall thickness in pulmonary arterioles. Scale bar: 50 μm (J) Masson staining of collagen around rat pulmonary arterioles. Scale bar: 20 μm (K) Comparison of the degree of pulmonary vascularized in rats of each group. Scale bar: 20 μm (L) Expression of OPN in the pulmonary vessels of rats in each group. Scale bar: 30 μm. Sham (n = 6), saline (n = 7), AAV1‐control vector group (n = 7) and AAV1‐KLF4‐shRNA group (n = 8) at the end of the therapeutic experiment. Multiple comparisons were performed by one‐way ANOVA with SNK‐q. *P < .05, **P < .01, ***P < .001

As shown in Figure 5A‐5C, RVSP and mRVP in the saline group and the AAV1‐control vector group were significantly higher than those in the sham group, which suggest that the model of pulmonary hypertension was constructed successfully. In the AAV1‐KLF4‐shRNA group, RVSP and mRVP were significantly lower than those in the saline group and the AAV1‐control vector group, indicating that therapeutic interventions were effective.

The dP/dt max and RV contractility index in the saline group and the AAV1‐control vector group were significantly higher than those in the sham group. In the AAV1‐KLF4‐shRNA group, dP/dt max and RV contractility index were significantly lower than those in the saline group and the AAV1‐control vector group. (Figure 5D, 5E).

#### 3.2.2 Indicators reflect the diastolic function of the right ventricle, left ventricular hemodynamic parameters and heart rate changes in the therapeutic experiment

The RVEDP, the dP/dt min and the right ventricle Tau were not significantly different among each group. (Figure_3_SuppInfo).

There was no significant difference between the LVESP and LVEDP. The heart rate in all groups was within 249‐340/ minute range, and the difference of heart rate between each group was not statistically significant, so the hemodynamic test data of each group were comparable. (Figure_4_SuppInfo).

#### 3.2.3 Changes of the level of right ventricular hypertrophy, pulmonary arteriole remodelling and muscularization in the therapeutic experiment

The indices of right ventricular hypertrophy, such as RV weight, Fulton 's index and RV/BW, were significantly higher in the saline group and the AAV1‐control vector group than in the sham group. The RV weight, Fulton 's index and RV/BW in the AAV1‐KLF4‐shRNA group were significantly lower than those in the saline group and the AAV1‐control vector group. (Figure 5F‐5H).

Compared with the sham group, the pulmonary small vascular wall of the saline group and the AAV1‐control vector group were thickened, and their smooth muscle of the middle layer was significantly proliferated. In the AAV1‐KLF4‐shRNA group, the thickening of the small vascular wall was significantly reduced compared with that in the saline group and the AAV1‐control vector group (Figure 5I). Similarly, Masson staining showed that the deposition of matrix protein around pulmonary vessels in model group was increased, which was significantly reduced after therapeutic intervention (Figure 5J).The muscularized degree of pulmonary vessels in the saline group and the AAV1‐control vector group was significantly higher than that in the sham group. The muscularized degree of pulmonary vessels in the AAV1‐KLF4‐shRNA group was significantly lower than that in the saline group and the AAV1‐control vector group (Figure 5K).

#### 3.2.4 Expression of OPN, KLF4 and number of PCNA‐positive cells in the pulmonary vessels in the therapeutic experiment

Immunohistochemical staining showed that the expression of OPN in the pulmonary vessels of the saline group and the AAV1‐control vector group was significantly stronger than that in the sham group, but the expression of OPN in the pulmonary vessels of the AAV1‐KLF4‐shRNA group was significantly weaker than that of the saline group and the AAV1‐control vector group (Figure 5L).

**FIGURE 6:**
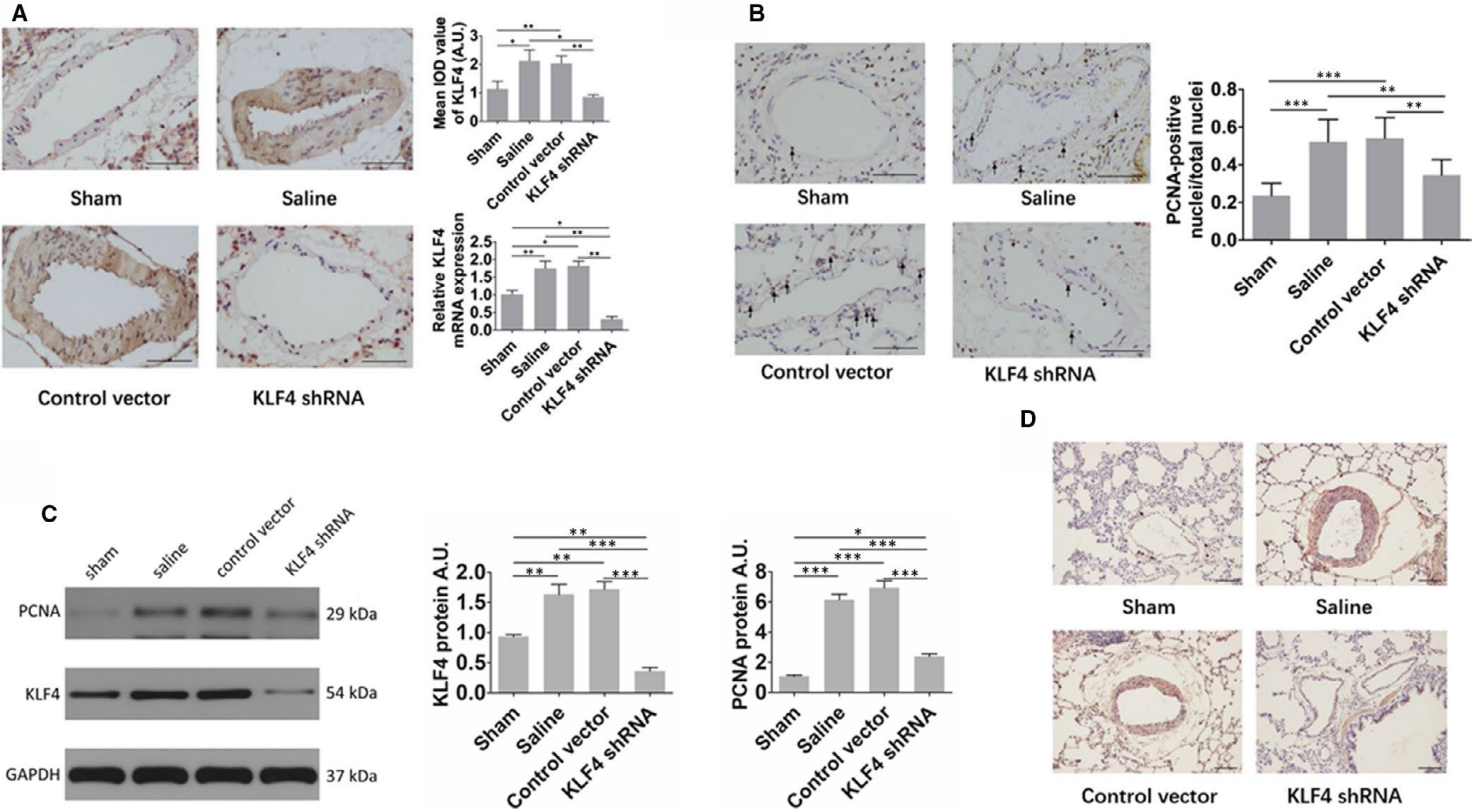
(A) Expression of KLF4 in the pulmonary vessels of rats in each group. Scale bar: 30 μm (B) Expression of PCNA in the pulmonary vascular smooth muscle of rats in each group. Scale bar: 25 μm (C) Representative bands of protein expression for KLF4 and PCNA in rat pulmonary vessels, and comparison of relative protein levels (normalized to GAPDH level). AU, arbitrary units. (D) Expression of P‐AKT in the pulmonary vessels of rats in each group. Scale bar: 20 μm. Sham (n = 6), saline (n = 7), AAV1‐control vector group (n = 7) and AAV1‐KLF4‐shRNA group (n = 8) at the end of the therapeutic experiment. Multiple comparisons were performed by one‐way ANOVA with SNK‐q. *P < .05, **P < .01, ***P < .001

The expression of KLF4 in the pulmonary vessels of the saline group and the AAV1‐control vector group was significantly stronger than that in the sham group, but the expression of KLF4 in the pulmonary vessels of the AAV1‐KLF4‐shRNA group was significantly weaker than that of the saline group and the AAV1‐control vector group (Figure 6A). We also detected the expression of KLF4 mRNA in 4 groups of rats by qRT‐PCR. The results showed that KLF4 in the two groups of PH models (the saline group and the AAV1‐control vector group) was significantly higher than that in healthy controls. The treatment intervention (AAV1‐KLF4‐shRNA) group was significantly lower than the other 3 groups (Figure 6A), indicating that the pulmonary vascular‐specific KLF4 knockdown effect is ideal in this experiment.

The immunohistochemical test of PCNA showed that the amount of PCNA‐positive smooth muscle cells in small lung vessels of the saline group and the AAV1‐control vector group appeared to be much more than that of the sham group,but the amount of PCNA‐positive smooth muscle cells in small lung vessels of the AAV1‐KLF4‐shRNA group was significantly less than that of the saline group and the AAV1‐control vector group (Figure 6B).

We also detected the protein expression for KLF4 and PCNA in rat pulmonary vessels by Western blot. The results were consistent with the trend of immunohistochemistry (Figure 6C).

#### 3.2.5 Changes of the expression of P‐AKT in the pulmonary vessels in the therapeutic experiment

We detected the expression of P‐AKT in pulmonary vessels of rats in each group. As shown in Figure 6D, The expression of P‐AKT in the pulmonary vessels of the saline group and the AAV1‐control vector group was significantly stronger than that in the sham group, but the expression in the pulmonary vessels of the AAV1‐KLF4‐shRNA group was significantly weaker than that of the saline group and the AAV1‐control vector group.

## 4 DISCUSSION

This research suggested that gene knockdown of KLF4 *via* intratracheal administration of AAV1‐KLF4‐shRNA had a preventive and therapeutic effects in vivo on CS‐induced PH rat model by inhibiting pulmonary vascular remodelling and improving right ventricular haemodynamics.

Some scientists have demonstrated that cigarette smoke could directly cause pulmonary vascular remodelling, and this process developed before the formation of emphysema and hypoxia.^8^ Our previous researches also confirmed this evidence.^9^, ^10^, ^11^ However, the underlying mechanism needs to be clarified. We reported previously that KLF4 expression levels were up‐regulated in lung vessel samples from rats with pulmonary vascular remodelling and in cultured proliferating human PASMCs.^14^ Gene silencing of KLF4 exerted beneficial effects by inhibiting the activation of the AKT pathway and, thereby, cell proliferation that resulted in near‐normal vessel morphology. KLF4 was considered to be closely involved in the pathogenesis of cigarette smoke‐induced PH. So, we observed the preventive and therapeutic effects of knockdown of KLF4 on CS‐induced PH rat model with recombinant AAV vector.

Recombinant AAV was considered as a popular vector for gene therapy because of its ability to achieve long and stable transgene expression, the fact that no human disease is caused by AAV infection, and the extremely low integration risk of AAV in the host genome.^25^ AAV1, one of the most efficient serotypes of AAV for gene transfection in vivo,^26^ has a selective affinity to blood vessels.^17^, ^27^, ^28^, ^29^ Taking advantage of this feature, we transfected AAV1 into the lung vessels as much as possible by intratracheal injection, in order to construct the pulmonary vascular‐specific KLF4 gene knockdown model. Our study provides the evidence that a successful AAV1‐based gene silencing intervention modulated progression of PH in an animal model. We provide data that support feasibility, efficiency and safety of airway distribution and transfection of small pulmonary vessels using an AAV1 vector as a novel delivery method for gene silencing in PH.

Herein, we show that a single intratracheal delivery of AAV1‐KLF4‐shRNA has preventive and therapeutic efficacy in PH. Five months after intratracheal delivery, the GFP expression was still detectable in the pulmonary tissue of animals transfected with AAV1‐KLF4‐shRNA or AAV1‐control vector, and it all distributed along the pulmonary vessels, indicating that the transfection effect is good. In addition, KLF4 expression was reduced in the AAV1‐KLF4‐shRNA group, demonstrating gene silencing was effective.

Our current research showed that the development of PH was improved by intratracheal administration of AAV1‐KLF4‐shRNA. This was proved by amelioration of the pulmonary haemodynamic indicators. These results also support pulmonary vessel remodelling measured by medial wall thickness of pulmonary arteries. We further proved that KLF4 was up‐regulated in the pulmonary arteries in the rat model of CS‐PH compared with the sham group. It is worth noting that pulmonary vessel KLF4 expression was significantly lower in AAV1‐KLF4‐shRNA‐treated rats compared with the saline group or AAV1‐control vector group, suggesting that there was efficient pulmonary vascular transduction and that reducing pulmonary artery KLF4 expression slowed the development of the disease. One research group previously reported a decreased expression of KLF4 in lungs from patients with pulmonary arterial hypertension (PAH) and demonstrated that KLF4 knockdown exacerbates PH in mice.^30^ It seems contradicted with our results. However, KLF4 is a transcription factor, which can regulate the transcriptional activation of target genes and has bidirectional regulation function.^31^ This is related to the different microenvironment and different signal pathways.^32^ In the study of primary breast ductal carcinoma and oral squamous cell carcinoma, KLF4 expression was increased and cell proliferation was promoted.^33^, ^34^ However, in some other tumours (such as oesophageal cancer, gastric cancer, medulloblastoma, bladder cancer, pancreatic cancer, colorectal cancer and pancreatic duct cancer), KLF4 plays a role in inhibiting the growth and proliferation of tumour cells.^35^, ^36^, ^37^, ^38^, ^39^, ^40^, ^41^, ^42^ The aetiology and pathogenesis of PH are complex. The pathogenesis and treatment of different types of PH are very different. Therefore, researchers need to select the appropriate modelling method according to the specific research purpose and the classification criteria of the disease. The PH caused by cigarette smoke in our study does not belong to the same category as the PAH they studied in pH classification, and their mechanism and method are naturally different. In addition, they specifically knocked down KLF4 in endothelial cells, which is also different from ours. In our previous study on PASMCs, we found that KLF4 can promote cell proliferation under the stimulation of CSE,^14^ which is consistent with our current in vivo experiments. Another team has come to a similar conclusion in vitro experiment with hypoxic PH.^43^

In the prevention experiment, the degree of pulmonary vascular remodelling, pulmonary arteriolar myogenesis, pulmonary vascular smooth muscle cell proliferation and right ventricular hypertrophy in the prevention group was significantly lower than those in the pH model group. The haemodynamic indexes of right ventricular systolic pressure, mean right ventricular pressure, the peak RV pressure rate of rise and right ventricular systolic index were significantly improved in the prevention group.

It has been reported that after 3 months of cigarette smoke stimulation, the pulmonary vascular remodelling in rats and the right ventricular systolic pressure increased significantly.^44^ Therefore, we selected smoking 3 months as the time‐point of treatment intervention in the treatment experiment. We found that the degree of pulmonary vascular remodelling, pulmonary arteriolar myogenesis, pulmonary vascular smooth muscle cell proliferation and right ventricular hypertrophy in the treatment group were significantly lower than those in the pH model group. The haemodynamic indexes of right ventricular systolic pressure, mean right ventricular pressure, the peak RV pressure rate of rise and right ventricular systolic index were also significantly improved in the treatment group.

The haemodynamic parameters of left ventricle did not change significantly. This shows that our method of modelling does not cause systemic circulation hypertension, and it also conforms to the expectation that the KLF4 gene in the blood vessels of the lung can be knocked down, and there is no effect on the circulation vessels. We also recorded the average heart rate during the experiments. The results showed no significant difference between each group, so the interference of heart rate difference on pulmonary circulation pressure was excluded.

**FIGURE 7:**
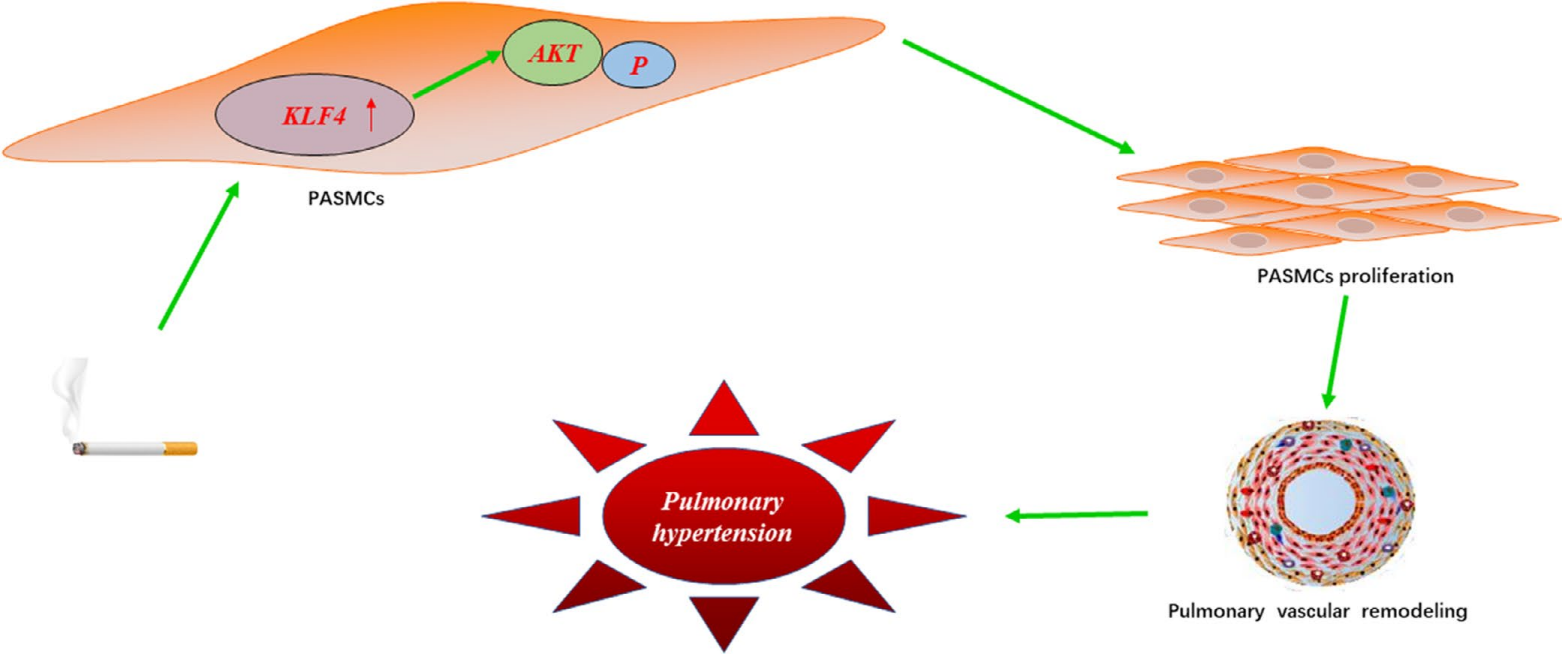
Schematic illustration of the potential role of KLF4 in the pathogenesis of cigarette smoke‐induced pulmonary hypertension

In our previous in vitro study, we found that KLF4 knockdown inhibited the CSE‐induced increase of AKT phosphorylation in PASMCs.^14^ In this in vivo study, we found that the expression of P‐AKT in pulmonary vessels of rats with pulmonary hypertension induced by cigarette smoke was significantly increased, while the expression of P‐AKT in pulmonary vessels of rats in AAV1‐KLF4‐shRNA group was significantly lower than that in model groups. So we believe that KLF4 plays a role in CS‐induced PH possibly via regulation of AKT signalling pathway, mediating its effect on PASMC proliferation and pulmonary vascular remodelling (Figure 7).

Our study has its own limitation. The sample size is relatively small, so this research may has limited power and limited follow‐up. Thus, further experiments about long‐term efficacy and safety end‐points are urgently needed prior to applying this airway gene‐silencing method to the clinic.

## 5 CONCLUSION

The present research proves that KLF4 plays an important role in CS‐induced PH, and selective pulmonary vascular gene knockdown of KLF4 with intratracheal instillation of AAV1‐KLF4‐shRNA ameliorates excessive vascular remodelling to lower pulmonary pressures. The results indicated that AAV1‐based gene intervention was efficacious in PH and position pulmonary vascular KLF4 gene knockdown via intratracheal instillation as a preventive and therapeutic modality in CS‐PH.

## CONFLICT OF INTEREST

The authors confirm that there are no conflicts of interest.

## AUTHOR CONTRIBUTIONS

Desheng Sun: Experimental design, manuscript writing and data analysis; DanDan Ding: Experiment modelling and data analysis; Qinghai Li: Experiment modelling and data collection; Min Xie: Data collection; Yongjian Xu: Data analysis; Xiansheng Liu: Experimental design and manuscript editing.

## Supporting information

Fig S1

Fig S2

Fig S3

Fig S4

Fig S5

Fig S6

## Data Availability

The data that support the findings of this study are available from the corresponding author upon reasonable request.
