# Supplementary figures and images for: The preventive and therapeutic effects of AAV1‐KLF4‐shRNA in cigarette smoke‐induced pulmonary hypertension
Source: J Cell Mol Med. 2020 Dec 20;25(2):1238–51. doi: 10.1111/jcmm.16194 (PMC7812256; doi:10.1111/jcmm.16194)

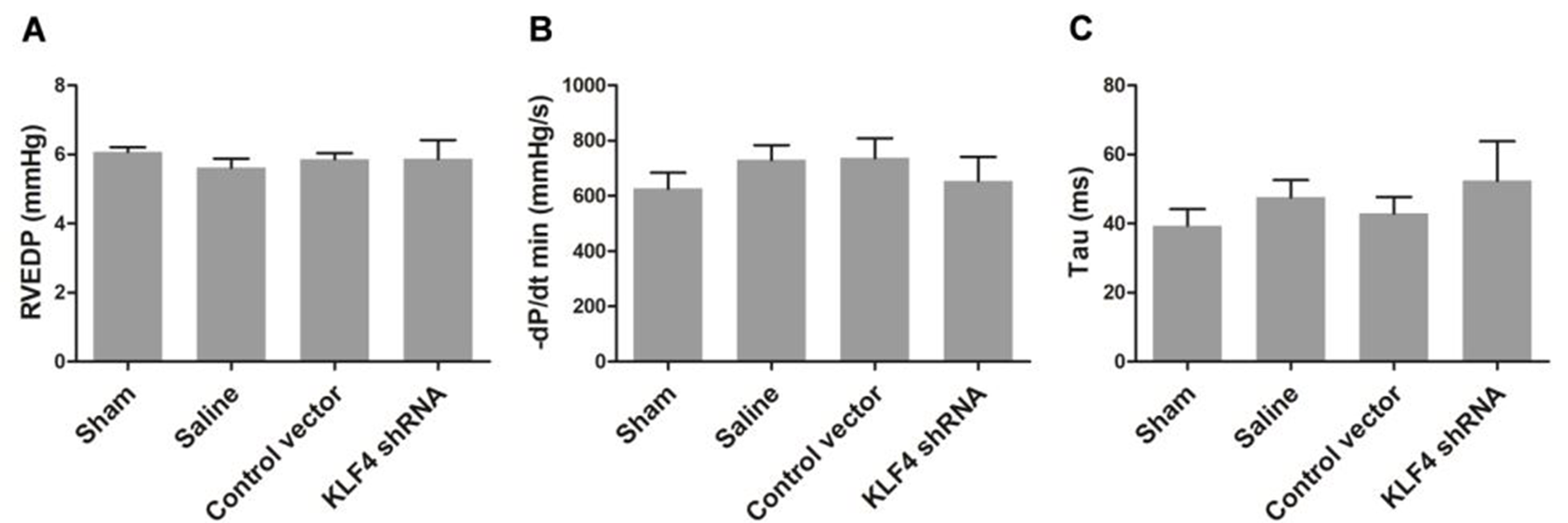

Supplement: Supplementary file 1 — Fig S1 [file JCMM-25-1238-s001.tif]

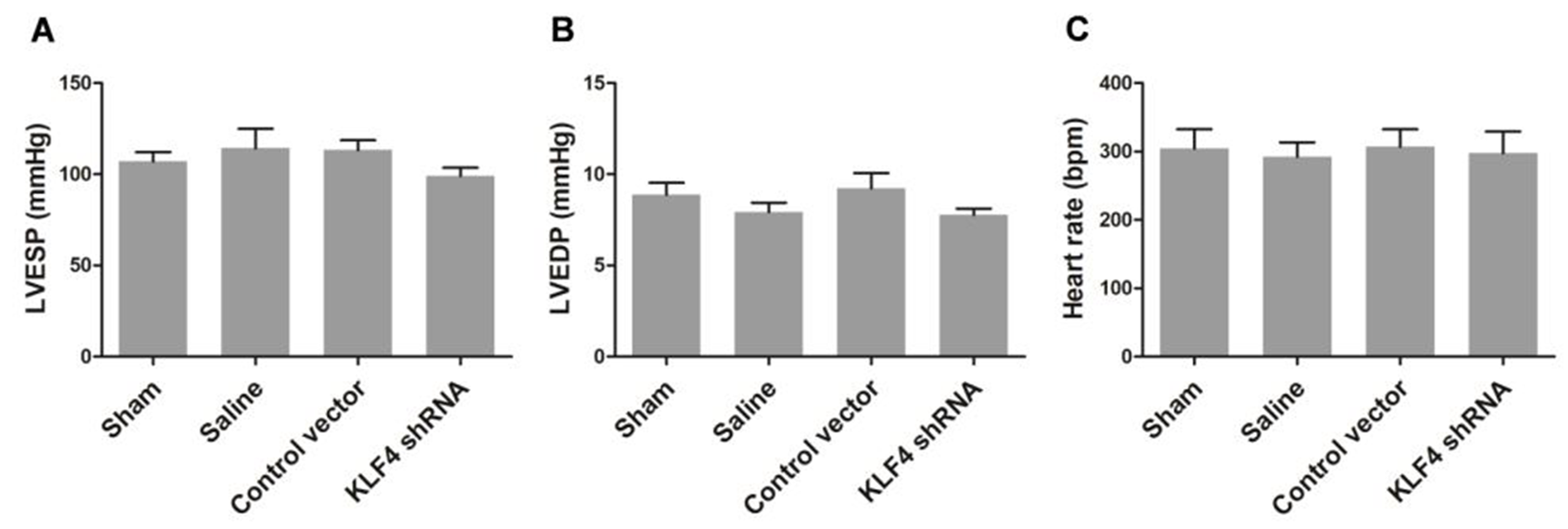

Supplement: Supplementary file 2 — Fig S2 [file JCMM-25-1238-s002.tif]

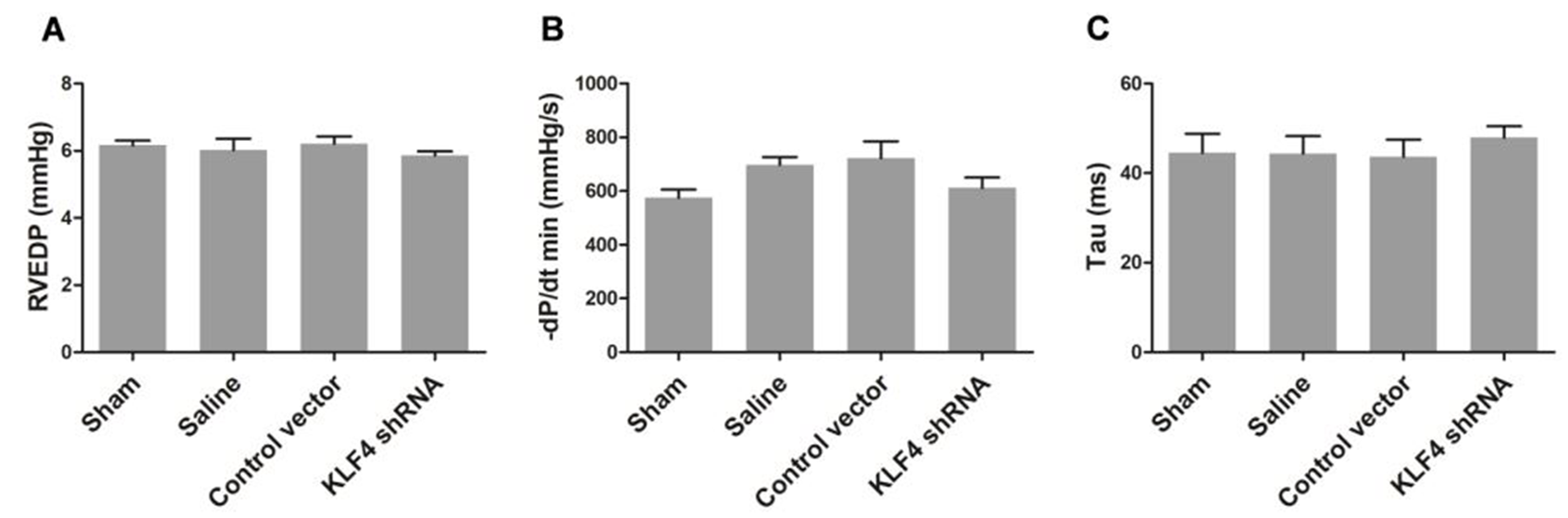

Supplement: Supplementary file 3 — Fig S3 [file JCMM-25-1238-s003.tif]

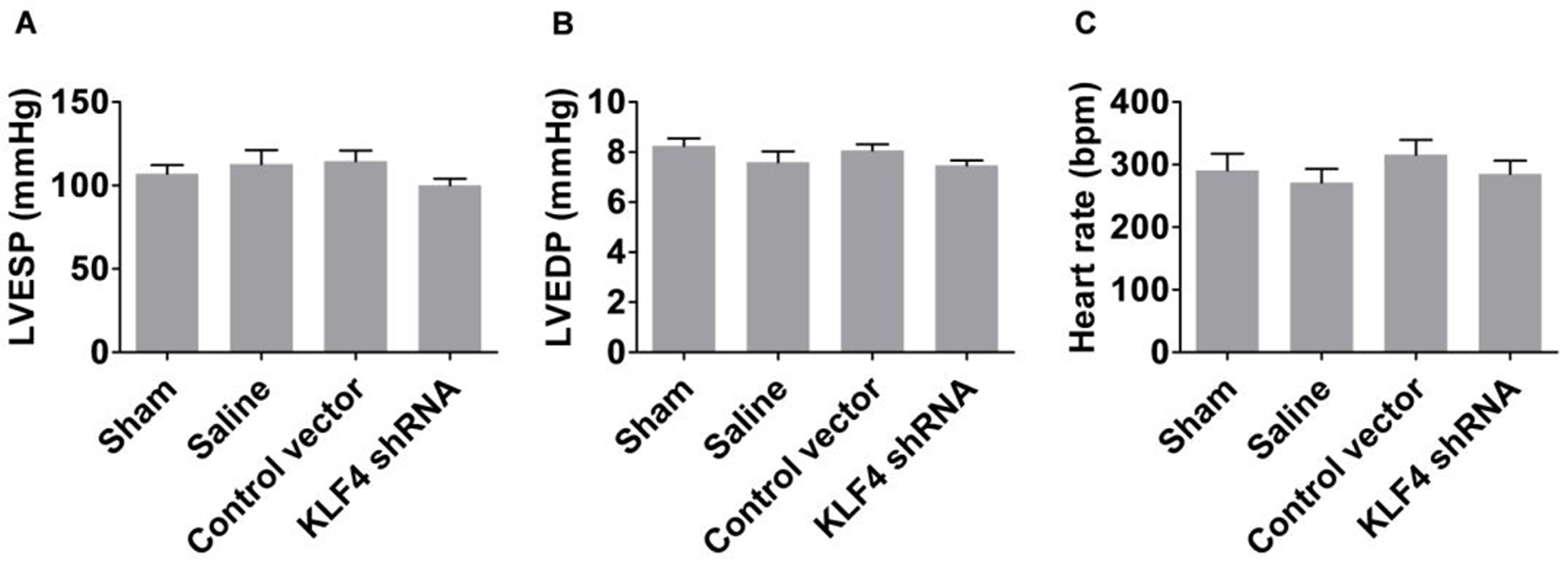

Supplement: Supplementary file 4 — Fig S4 [file JCMM-25-1238-s004.tif]

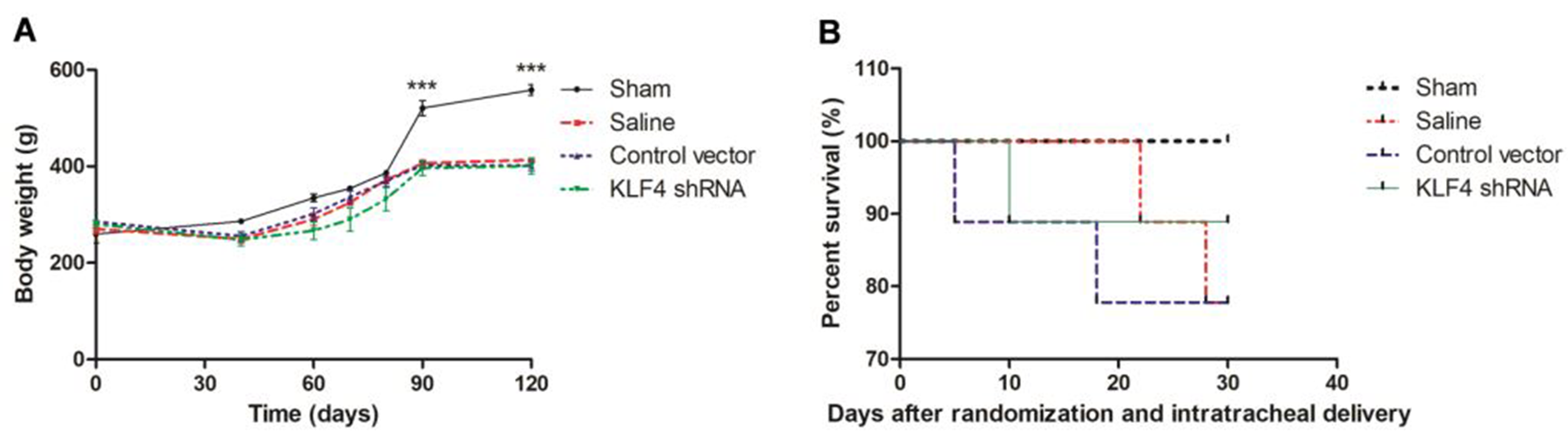

Supplement: Supplementary file 5 — Fig S5 [file JCMM-25-1238-s005.tif]

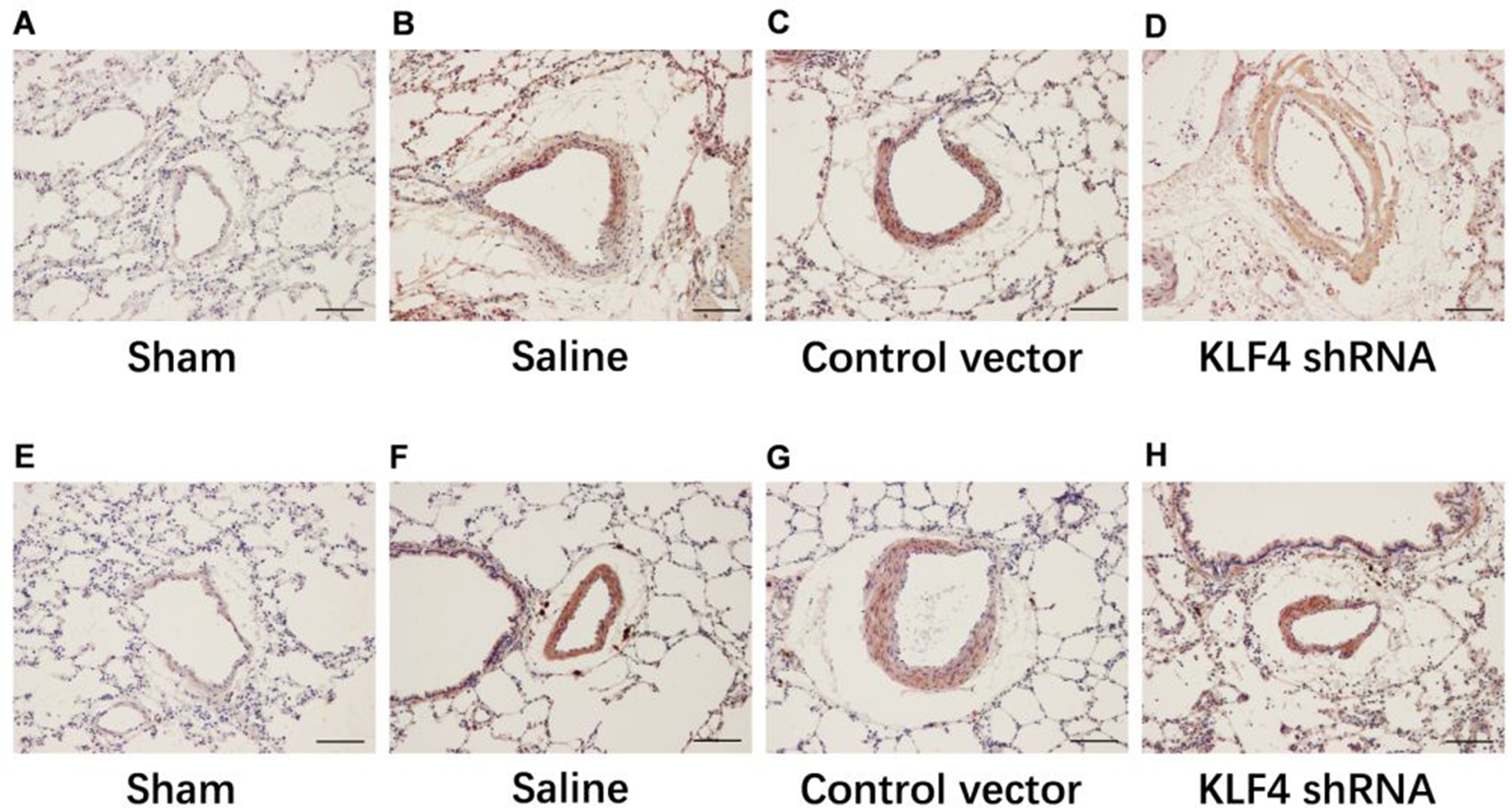

Supplement: Supplementary file 6 — Fig S6 [file JCMM-25-1238-s006.tif]
